# Bis(1-benzyl-1*H*-benzimidazole-κ*N* ^3^)di­chlorido­zinc

**DOI:** 10.1107/S1600536814002840

**Published:** 2014-02-12

**Authors:** Rachid Bouhfid, El Mokhtar Essassi, Mohamed Saadi, Lahcen El Ammari

**Affiliations:** a Centre Composites Nanocomposites, Moroccan Foundation for Advanced Science, Innovation and Research (MAScIR), Rabat Design Center, Rue Mohamed Al Jazouli-Madinat Al Irfane, Rabat 10100, Morocco; b Laboratoire de Chimie Organique Hétérocyclique, URAC 21, Pôle de Compétences Pharmacochimie, Université Mohammed V-Agdal, Avenue Ibn Battouta, BP 1014, Rabat, Morocco; c Laboratoire de Chimie du Solide Appliquée, Faculté des Sciences, Université Mohammed V-Agdal, Avenue Ibn Battouta, BP 1014, Rabat, Morocco

## Abstract

In the title compound, [ZnCl_2_(C_14_H_12_N_2_)_2_], the Zn^II^ atom exhibits a distorted tetra­hedral coordination geometry involving two chloride anions and two N-atom donors from 1-benzyl-1*H*-benzimidazole ligands. In both ligands, the benzyl and benzimidazole rings are nearly perpendicular [dihedral angles = 81.7 (2) and 81.5 (2)°]. The two benzimidazole systems are essentially planar [maximum deviations = 0.015 (3) and 0.020 (2) Å] and form a dihedral angle of 78.09 (8)°. In the crystal, centrosymmetrically related mol­ecules are linked by pairs of C—H⋯Cl hydrogen bonds into chains parallel to the *a* axis.

## Related literature   

For background to the biochemical properties of benzimidazole derivatives, see: Mann *et al.* (2001 ▶); Naithani *et al.* (1990 ▶); Goudgaon *et al.* (2004 ▶). For the structures of related compounds see: Abdel-Ghani & Mansour (2011 ▶, 2012 ▶); Ahuja & Prasad (1976 ▶).
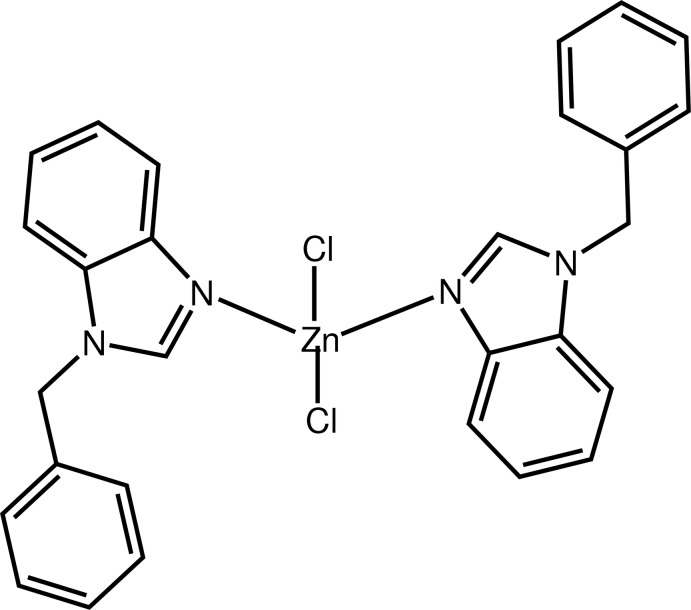


## Experimental   

### 

#### Crystal data  

[ZnCl_2_(C_14_H_12_N_2_)_2_]
*M* *_r_* = 552.78
Triclinic, 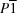

*a* = 9.9819 (4) Å
*b* = 10.0564 (4) Å
*c* = 13.6641 (6) Å
α = 99.539 (2)°
β = 92.087 (2)°
γ = 99.122 (2)°
*V* = 1332.79 (10) Å^3^
*Z* = 2
Mo *K*α radiation
μ = 1.15 mm^−1^
*T* = 296 K
0.37 × 0.32 × 0.26 mm

#### Data collection  

Bruker X8 APEX diffractometer
Absorption correction: multi-scan (*SADABS*; Bruker, 2009 ▶) *T*_min_ = 0.682, *T*_max_ = 0.840
30968 measured reflections
7403 independent reflections
5928 reflections with *I* > 2σ(*I*)
*R*_int_ = 0.026

#### Refinement  

*R*[*F* ^2^ > 2σ(*F* ^2^)] = 0.039
*wR*(*F* ^2^) = 0.108
*S* = 1.06
7403 reflections
318 parameters
H-atom parameters constrained
Δρ_max_ = 0.58 e Å^−3^
Δρ_min_ = −0.24 e Å^−3^

### 

Data collection: *APEX2* (Bruker, 2009 ▶); cell refinement: *SAINT* (Bruker, 2009 ▶); data reduction: *SAINT*; program(s) used to solve structure: *SHELXS97* (Sheldrick, 2008 ▶); program(s) used to refine structure: *SHELXL97* (Sheldrick, 2008 ▶); molecular graphics: *ORTEP-3 for Windows* (Farrugia, 2012 ▶); software used to prepare material for publication: *PLATON* (Spek, 2009 ▶) and *publCIF* (Westrip, 2010 ▶).

## Supplementary Material

Crystal structure: contains datablock(s) I. DOI: 10.1107/S1600536814002840/rz5104sup1.cif

Structure factors: contains datablock(s) I. DOI: 10.1107/S1600536814002840/rz5104Isup2.hkl

CCDC reference:

Additional supporting information:  crystallographic information; 3D view; checkCIF report

## Figures and Tables

**Table 1:** Hydrogen-bond geometry (Å, °)

*D*—H⋯*A*	*D*—H	H⋯*A*	*D*⋯*A*	*D*—H⋯*A*
C14—H14⋯Cl1^i^	0.93	2.81	3.660 (2)	153
C28—H28⋯Cl1^ii^	0.93	2.80	3.502 (2)	133

Symmetry codes: (i) 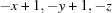; (ii) 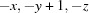.

**Fig. 1.**
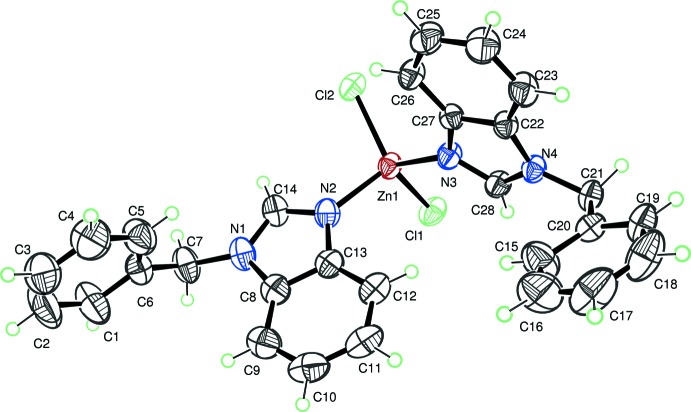
The molecular structure of the title compound with displacement ellipsoids drawn at the 50% probability level. H atoms are represented as small circles.

## supplementary crystallographic information

### 1. Comment

Benzimidazole derivatives are important pharmacophores in drug discovery, possessing pharmacological properties including antitumor (Mann *et al.*, 2001), anti-Parkinson (Naithani *et al.*, 1990) and antimicrobial (Goudgaon *et al.*, 2004) activities. A considerable number of metal benzimidazole complexes including Cr, Mn, Fe, Co, Ni, Zn, Pd, Pt, Au, and Re has been reported (Abdel-Ghani & Mansour, 2011, 2012). Metal complexes of biologically important ligands were sometimes more effective than the free ligands (Ahuja & Prasad, 1976).

The crystal structure of the title compound, show that the Zn^II^ ion adopts a distorted tetrahedral coordination arising from two N-atom donors from organic ligands and a two Cl^-^ anions (Fig. 1). The fused five- and six-membered rings are nearly coplanar with dihedral angles between them of 1.0 (2)° and 1.6 (2)° respectively. The dihedral angle between the two benzimidazole systems is of 78.09 (8)°. Each benzyl ring (C1–C6 and C15–C20) is virtually perpendicular to the benzimidazole system belonging to the same molecule (N1/N2/C8–C14 and N3/N4/C22–C28) as indicated by the dihedral angles between them of 81.7 (2)° and 81.5 (2)°, respectively. In the crystal, centrosymmetrically-related molecules are linked by C—H···Cl hydrogen bonds (Table 1) into chains running parallel to the *a* axis.

### 2. Experimental

The title compound was prepared by the reaction of ZnCl_2_ (164 mg, 1.2 mmol) in water (10 ml) and 1-benzyl-1*H*-benzimidazole (500 mg, 2.4 mmol) in ethanol (10 ml). The mixture was stirred for 12 h, filtered and set aside to crystallize at ambient temperature for several days, giving colourless single crystals on slow evaporation of the solvent.

### 3. Refinement

H atoms were located in a difference Fourier map and treated as riding with C—H = 0.93–0.97 Å (methylene), and with *U*_iso_(H) = 1.2 *U*_eq_(C). Three outliers (0 0 1, 1 0 0, 0 1 0) were omitted in the last cycles of refinement.

### Crystal data

**Table:**

[ZnCl_2_(C_14_H_12_N_2_)_2_]	*V* = 1332.79 (10) Å^3^
*M**_r_* = 552.78	*Z* = 2
Triclinic, *P*1	*F*(000) = 568
Hall symbol: -p 1	*D*_x_ = 1.377 Mg m^−^^3^
*a* = 9.9819 (4) Å	Mo *K*α radiation, λ = 0.71073 Å
*b* = 10.0564 (4) Å	µ = 1.15 mm^−^^1^
*c* = 13.6641 (6) Å	*T* = 296 K
α = 99.539 (2)°	Block, colourless
β = 92.087 (2)°	0.37 × 0.32 × 0.26 mm
γ = 99.122 (2)°	

### Data collection

**Table:**

Bruker X8 APEX diffractometer	7403 independent reflections
Radiation source: fine-focus sealed tube	5928 reflections with *I* > 2σ(*I*)
Graphite monochromator	*R*_int_ = 0.026
φ and ω scans	θ_max_ = 29.6°, θ_min_ = 2.4°
Absorption correction: multi-scan (*SADABS*; Bruker, 2009)	*h* = −13→13
*T*_min_ = 0.682, *T*_max_ = 0.840	*k* = −13→13
30968 measured reflections	*l* = −18→18

### Refinement

**Table:**

Refinement on *F*^2^	Primary atom site location: structure-invariant direct methods
Least-squares matrix: full	Secondary atom site location: difference Fourier map
*R*[*F*^2^ > 2σ(*F*^2^)] = 0.039	Hydrogen site location: inferred from neighbouring sites
*wR*(*F*^2^) = 0.108	H-atom parameters constrained
*S* = 1.06	*w* = 1/[σ^2^(*F*_o_^2^) + (0.0551*P*)^2^ + 0.3641*P*] where *P* = (*F*_o_^2^ + 2*F*_c_^2^)/3
7403 reflections	(Δ/σ)_max_ < 0.001
318 parameters	Δρ_max_ = 0.58 e Å^−^^3^
0 restraints	Δρ_min_ = −0.24 e Å^−^^3^

### Special details

**Table:**

Geometry. All e.s.d.'s (except the e.s.d. in the dihedral angle between two l.s. planes) are estimated using the full covariance matrix. The cell e.s.d.'s are taken into account individually in the estimation of e.s.d.'s in distances, angles and torsion angles; correlations between e.s.d.'s in cell parameters are only used when they are defined by crystal symmetry. An approximate (isotropic) treatment of cell e.s.d.'s is used for estimating e.s.d.'s involving l.s. planes.
Refinement. Refinement of *F*^2^ against all reflections. The weighted *R*-factor *wR* and goodness of fit *S* are based on *F*^2^, conventional *R*-factors *R* are based on *F*, with *F* set to zero for negative *F*^2^. The threshold expression of *F*^2^ > σ(*F*^2^) is used only for calculating *R*-factors(gt) *etc*. and is not relevant to the choice of reflections for refinement. *R*-factors based on *F*^2^ are statistically about twice as large as those based on *F*, and *R*- factors based on all data will be even larger.

### Fractional atomic coordinates and isotropic or equivalent isotropic displacement parameters (Å^2^)

**Table:**

	*x*	*y*	*z*	*U*_iso_*/*U*_eq_	
C1	0.7633 (4)	0.8525 (3)	0.4366 (3)	0.1008 (11)	
H1	0.7791	0.9301	0.4072	0.121*	
C2	0.8206 (5)	0.8556 (4)	0.5311 (3)	0.1210 (14)	
H2	0.8754	0.9350	0.5641	0.145*	
C3	0.7979 (4)	0.7450 (5)	0.5757 (2)	0.1088 (13)	
H3	0.8379	0.7477	0.6387	0.131*	
C4	0.7162 (4)	0.6296 (4)	0.5281 (3)	0.1052 (12)	
H4	0.6985	0.5537	0.5593	0.126*	
C5	0.6591 (4)	0.6246 (3)	0.4331 (2)	0.0826 (8)	
H5	0.6036	0.5451	0.4010	0.099*	
C6	0.6835 (2)	0.7356 (2)	0.38635 (17)	0.0602 (5)	
C7	0.6298 (3)	0.7325 (3)	0.28114 (18)	0.0675 (6)	
H7A	0.6199	0.8247	0.2733	0.085 (9)*	
H7B	0.6962	0.7031	0.2359	0.084 (9)*	
C8	0.3754 (2)	0.6587 (2)	0.29074 (15)	0.0539 (5)	
C9	0.3381 (3)	0.7496 (3)	0.36933 (17)	0.0692 (6)	
H9	0.4018	0.8181	0.4071	0.083*	
C10	0.2033 (4)	0.7334 (3)	0.3883 (2)	0.0799 (8)	
H10	0.1754	0.7914	0.4411	0.096*	
C11	0.1069 (3)	0.6330 (3)	0.3312 (2)	0.0775 (7)	
H11	0.0163	0.6255	0.3465	0.093*	
C12	0.1428 (3)	0.5448 (3)	0.25247 (19)	0.0644 (6)	
H12	0.0780	0.4784	0.2138	0.077*	
C13	0.2792 (2)	0.5583 (2)	0.23266 (15)	0.0510 (4)	
C14	0.4755 (2)	0.5385 (2)	0.17551 (15)	0.0538 (5)	
H14	0.5432	0.5085	0.1369	0.065*	
C15	−0.2428 (3)	0.2655 (3)	0.2558 (2)	0.0821 (8)	
H15	−0.1617	0.3181	0.2437	0.099*	
C16	−0.2920 (5)	0.2837 (4)	0.3495 (2)	0.1078 (12)	
H16	−0.2421	0.3458	0.4010	0.129*	
C17	−0.4137 (4)	0.2106 (5)	0.3665 (2)	0.1047 (13)	
H17	−0.4492	0.2271	0.4285	0.126*	
C18	−0.4834 (3)	0.1131 (4)	0.2925 (2)	0.0917 (10)	
H18	−0.5648	0.0613	0.3049	0.110*	
C19	−0.4329 (2)	0.0915 (3)	0.19898 (18)	0.0638 (6)	
H19	−0.4797	0.0243	0.1490	0.077*	
C20	−0.3139 (2)	0.1694 (2)	0.18024 (15)	0.0487 (4)	
C21	−0.26224 (18)	0.1483 (2)	0.07709 (15)	0.0491 (4)	
H21A	−0.3058	0.0600	0.0407	0.059*	
H21B	−0.2862	0.2182	0.0418	0.059*	
C22	−0.04380 (18)	0.06922 (18)	0.12376 (13)	0.0409 (4)	
C23	−0.0871 (2)	−0.0469 (2)	0.16412 (17)	0.0570 (5)	
H23	−0.1788	−0.0802	0.1678	0.068*	
C24	0.0132 (3)	−0.1098 (2)	0.1982 (2)	0.0688 (6)	
H24	−0.0113	−0.1878	0.2261	0.083*	
C25	0.1510 (3)	−0.0600 (2)	0.19231 (18)	0.0638 (6)	
H25	0.2157	−0.1056	0.2166	0.077*	
C26	0.1940 (2)	0.0551 (2)	0.15140 (15)	0.0510 (4)	
H26	0.2858	0.0878	0.1472	0.061*	
C27	0.09351 (18)	0.11926 (18)	0.11694 (12)	0.0393 (3)	
C28	−0.02314 (18)	0.24853 (19)	0.05074 (14)	0.0447 (4)	
H28	−0.0461	0.3176	0.0191	0.054*	
N1	0.49942 (19)	0.6426 (2)	0.25291 (13)	0.0562 (4)	
N2	0.34613 (17)	0.48349 (18)	0.16007 (13)	0.0515 (4)	
N3	0.10356 (15)	0.23339 (16)	0.07047 (12)	0.0443 (3)	
N4	−0.11488 (15)	0.15456 (16)	0.08096 (12)	0.0425 (3)	
Zn1	0.26873 (2)	0.35268 (2)	0.034848 (16)	0.04370 (8)	
Cl1	0.18906 (5)	0.46699 (5)	−0.07679 (4)	0.05779 (14)	
Cl2	0.43183 (5)	0.23659 (6)	−0.01957 (4)	0.05514 (13)	

### Atomic displacement parameters (Å^2^)

**Table:**

	*U*^11^	*U*^22^	*U*^33^	*U*^12^	*U*^13^	*U*^23^
C1	0.128 (3)	0.0754 (18)	0.082 (2)	−0.0178 (19)	−0.027 (2)	0.0067 (16)
C2	0.147 (4)	0.105 (3)	0.085 (2)	−0.009 (2)	−0.049 (2)	−0.015 (2)
C3	0.131 (3)	0.130 (3)	0.0621 (17)	0.044 (3)	−0.0313 (19)	−0.005 (2)
C4	0.139 (3)	0.101 (2)	0.081 (2)	0.032 (2)	−0.024 (2)	0.0289 (19)
C5	0.105 (2)	0.0691 (16)	0.0702 (16)	0.0086 (15)	−0.0213 (15)	0.0133 (13)
C6	0.0603 (13)	0.0638 (13)	0.0514 (11)	0.0031 (10)	−0.0037 (10)	0.0035 (10)
C7	0.0673 (14)	0.0725 (15)	0.0551 (12)	−0.0147 (12)	−0.0033 (11)	0.0164 (11)
C8	0.0656 (13)	0.0573 (11)	0.0423 (10)	0.0116 (10)	−0.0011 (9)	0.0186 (8)
C9	0.0940 (19)	0.0659 (14)	0.0497 (12)	0.0184 (13)	−0.0031 (12)	0.0124 (10)
C10	0.107 (2)	0.0841 (18)	0.0608 (14)	0.0472 (17)	0.0141 (15)	0.0157 (13)
C11	0.0728 (16)	0.094 (2)	0.0791 (17)	0.0406 (15)	0.0165 (14)	0.0269 (15)
C12	0.0562 (12)	0.0737 (15)	0.0688 (14)	0.0189 (11)	0.0059 (11)	0.0198 (12)
C13	0.0550 (11)	0.0539 (11)	0.0479 (10)	0.0116 (9)	0.0019 (8)	0.0177 (8)
C14	0.0516 (11)	0.0620 (12)	0.0454 (10)	−0.0021 (9)	0.0040 (8)	0.0136 (9)
C15	0.090 (2)	0.0796 (18)	0.0657 (15)	−0.0004 (15)	0.0121 (14)	−0.0051 (13)
C16	0.128 (3)	0.124 (3)	0.0638 (18)	0.033 (3)	0.0048 (19)	−0.0162 (18)
C17	0.099 (2)	0.178 (4)	0.0517 (15)	0.064 (3)	0.0180 (16)	0.0198 (19)
C18	0.0571 (15)	0.161 (3)	0.0696 (17)	0.0273 (18)	0.0195 (13)	0.045 (2)
C19	0.0406 (10)	0.0944 (17)	0.0597 (12)	0.0133 (11)	0.0059 (9)	0.0199 (12)
C20	0.0455 (10)	0.0543 (11)	0.0502 (10)	0.0158 (8)	0.0057 (8)	0.0124 (8)
C21	0.0338 (8)	0.0610 (11)	0.0525 (10)	0.0051 (8)	0.0037 (7)	0.0129 (9)
C22	0.0399 (8)	0.0422 (9)	0.0400 (8)	0.0031 (7)	−0.0006 (7)	0.0099 (7)
C23	0.0510 (11)	0.0547 (11)	0.0668 (13)	−0.0035 (9)	0.0029 (9)	0.0267 (10)
C24	0.0727 (15)	0.0575 (13)	0.0822 (16)	0.0032 (11)	−0.0030 (12)	0.0387 (12)
C25	0.0673 (14)	0.0640 (13)	0.0676 (14)	0.0189 (11)	−0.0075 (11)	0.0279 (11)
C26	0.0440 (10)	0.0595 (11)	0.0519 (10)	0.0106 (8)	−0.0034 (8)	0.0160 (9)
C27	0.0400 (8)	0.0402 (8)	0.0375 (8)	0.0043 (7)	−0.0002 (6)	0.0092 (7)
C28	0.0408 (9)	0.0429 (9)	0.0539 (10)	0.0070 (7)	0.0038 (8)	0.0182 (8)
N1	0.0570 (10)	0.0648 (11)	0.0434 (8)	−0.0043 (8)	−0.0011 (7)	0.0144 (8)
N2	0.0464 (9)	0.0572 (10)	0.0502 (9)	0.0023 (7)	0.0031 (7)	0.0129 (7)
N3	0.0377 (7)	0.0442 (8)	0.0539 (9)	0.0042 (6)	0.0031 (6)	0.0189 (7)
N4	0.0359 (7)	0.0451 (8)	0.0479 (8)	0.0051 (6)	0.0031 (6)	0.0140 (6)
Zn1	0.03618 (11)	0.04504 (13)	0.05179 (14)	0.00320 (8)	0.00320 (8)	0.01722 (9)
Cl1	0.0506 (3)	0.0594 (3)	0.0722 (3)	0.0134 (2)	0.0037 (2)	0.0330 (3)
Cl2	0.0428 (2)	0.0630 (3)	0.0643 (3)	0.0156 (2)	0.0044 (2)	0.0178 (2)

### Geometric parameters (Å, º)

**Table:**

C1—C6	1.373 (4)	C15—H15	0.9300
C1—C2	1.386 (5)	C16—C17	1.365 (6)
C1—H1	0.9300	C16—H16	0.9300
C2—C3	1.347 (6)	C17—C18	1.368 (5)
C2—H2	0.9300	C17—H17	0.9300
C3—C4	1.359 (5)	C18—C19	1.387 (4)
C3—H3	0.9300	C18—H18	0.9300
C4—C5	1.388 (4)	C19—C20	1.372 (3)
C4—H4	0.9300	C19—H19	0.9300
C5—C6	1.370 (4)	C20—C21	1.512 (3)
C5—H5	0.9300	C21—N4	1.461 (2)
C6—C7	1.509 (3)	C21—H21A	0.9700
C7—N1	1.461 (3)	C21—H21B	0.9700
C7—H7A	0.9700	C22—N4	1.382 (2)
C7—H7B	0.9700	C22—C23	1.387 (3)
C8—N1	1.381 (3)	C22—C27	1.395 (2)
C8—C9	1.393 (3)	C23—C24	1.372 (3)
C8—C13	1.394 (3)	C23—H23	0.9300
C9—C10	1.369 (4)	C24—C25	1.398 (4)
C9—H9	0.9300	C24—H24	0.9300
C10—C11	1.389 (4)	C25—C26	1.382 (3)
C10—H10	0.9300	C25—H25	0.9300
C11—C12	1.373 (4)	C26—C27	1.384 (3)
C11—H11	0.9300	C26—H26	0.9300
C12—C13	1.387 (3)	C27—N3	1.393 (2)
C12—H12	0.9300	C28—N3	1.321 (2)
C13—N2	1.398 (3)	C28—N4	1.336 (2)
C14—N2	1.316 (3)	C28—H28	0.9300
C14—N1	1.344 (3)	N2—Zn1	2.0225 (17)
C14—H14	0.9300	N3—Zn1	2.0048 (15)
C15—C20	1.380 (3)	Zn1—Cl2	2.2292 (5)
C15—C16	1.382 (4)	Zn1—Cl1	2.2503 (5)
			
C6—C1—C2	120.4 (3)	C17—C18—C19	120.1 (3)
C6—C1—H1	119.8	C17—C18—H18	120.0
C2—C1—H1	119.8	C19—C18—H18	120.0
C3—C2—C1	120.9 (3)	C20—C19—C18	120.0 (3)
C3—C2—H2	119.6	C20—C19—H19	120.0
C1—C2—H2	119.6	C18—C19—H19	120.0
C2—C3—C4	119.6 (3)	C19—C20—C15	119.6 (2)
C2—C3—H3	120.2	C19—C20—C21	119.6 (2)
C4—C3—H3	120.2	C15—C20—C21	120.8 (2)
C3—C4—C5	120.2 (3)	N4—C21—C20	111.42 (16)
C3—C4—H4	119.9	N4—C21—H21A	109.3
C5—C4—H4	119.9	C20—C21—H21A	109.3
C6—C5—C4	120.7 (3)	N4—C21—H21B	109.3
C6—C5—H5	119.6	C20—C21—H21B	109.3
C4—C5—H5	119.6	H21A—C21—H21B	108.0
C5—C6—C1	118.3 (3)	N4—C22—C23	131.75 (17)
C5—C6—C7	122.6 (2)	N4—C22—C27	105.86 (15)
C1—C6—C7	119.2 (2)	C23—C22—C27	122.35 (17)
N1—C7—C6	114.19 (19)	C24—C23—C22	116.2 (2)
N1—C7—H7A	108.7	C24—C23—H23	121.9
C6—C7—H7A	108.7	C22—C23—H23	121.9
N1—C7—H7B	108.7	C23—C24—C25	121.9 (2)
C6—C7—H7B	108.7	C23—C24—H24	119.0
H7A—C7—H7B	107.6	C25—C24—H24	119.0
N1—C8—C9	132.6 (2)	C26—C25—C24	121.8 (2)
N1—C8—C13	106.03 (19)	C26—C25—H25	119.1
C9—C8—C13	121.4 (2)	C24—C25—H25	119.1
C10—C9—C8	116.9 (3)	C25—C26—C27	116.63 (19)
C10—C9—H9	121.6	C25—C26—H26	121.7
C8—C9—H9	121.6	C27—C26—H26	121.7
C9—C10—C11	122.0 (3)	C26—C27—N3	130.31 (17)
C9—C10—H10	119.0	C26—C27—C22	121.08 (17)
C11—C10—H10	119.0	N3—C27—C22	108.59 (15)
C12—C11—C10	121.3 (3)	N3—C28—N4	113.13 (16)
C12—C11—H11	119.4	N3—C28—H28	123.4
C10—C11—H11	119.4	N4—C28—H28	123.4
C11—C12—C13	117.7 (3)	C14—N1—C8	107.04 (18)
C11—C12—H12	121.2	C14—N1—C7	125.4 (2)
C13—C12—H12	121.2	C8—N1—C7	127.1 (2)
C12—C13—C8	120.7 (2)	C14—N2—C13	105.48 (18)
C12—C13—N2	130.8 (2)	C14—N2—Zn1	123.26 (15)
C8—C13—N2	108.46 (19)	C13—N2—Zn1	129.57 (14)
N2—C14—N1	113.0 (2)	C28—N3—C27	105.30 (14)
N2—C14—H14	123.5	C28—N3—Zn1	124.79 (12)
N1—C14—H14	123.5	C27—N3—Zn1	129.89 (12)
C20—C15—C16	120.0 (3)	C28—N4—C22	107.12 (15)
C20—C15—H15	120.0	C28—N4—C21	126.89 (16)
C16—C15—H15	120.0	C22—N4—C21	125.84 (15)
C17—C16—C15	120.2 (3)	N3—Zn1—N2	107.29 (7)
C17—C16—H16	119.9	N3—Zn1—Cl2	113.31 (5)
C15—C16—H16	119.9	N2—Zn1—Cl2	107.21 (5)
C16—C17—C18	120.1 (3)	N3—Zn1—Cl1	104.56 (5)
C16—C17—H17	119.9	N2—Zn1—Cl1	110.42 (5)
C18—C17—H17	119.9	Cl2—Zn1—Cl1	113.88 (2)

### Hydrogen-bond geometry (Å, º)

**Table:**

*D*—H···*A*	*D*—H	H···*A*	*D*···*A*	*D*—H···*A*
C14—H14···Cl1^i^	0.93	2.81	3.660 (2)	153
C28—H28···Cl1^ii^	0.93	2.80	3.502 (2)	133

Symmetry codes: (i) −*x*+1, −*y*+1, −*z*; (ii) −*x*, −*y*+1, −*z*.
